# Reduced clinical and postmortem measures of cardiac pathology in subjects with advanced Alzheimer's Disease

**DOI:** 10.1186/1471-2318-11-3

**Published:** 2011-01-25

**Authors:** Thomas G Beach, Chera L Maarouf, Reed G Brooks, Scophil Shirohi, Ian D Daugs, Lucia I Sue, Marwan N Sabbagh, Douglas G Walker, LihFen Lue, Alex E Roher

**Affiliations:** 1 Banner Sun Health Research Institute, Sun City, AZ, USA

## Abstract

**Background:**

Epidemiological studies indicate a statistical linkage between atherosclerotic vascular disease (ATH) and Alzheimer's disease (AD). Autopsy studies of cardiac disease in AD have been few and inconclusive. In this report, clinical and gross anatomic measures of cardiac disease were compared in deceased human subjects with and without AD.

**Methods:**

Clinically documented cardiovascular conditions from AD (n = 35) and elderly non-demented control subjects (n = 22) were obtained by review of medical records. Coronary artery stenosis and other gross anatomical measures, including heart weight, ventricular wall thickness, valvular circumferences, valvular calcifications and myocardial infarct number and volume were determined at autopsy.

**Results:**

Compared to non-demented age-similar control subjects, those with AD had significantly fewer total diagnosed clinical conditions (2.91 vs 4.18), decreased coronary artery stenosis (70.8 vs 74.8%), heart weight (402 vs 489 g for males; 319 vs 412 g for females) and valvular circumferences. Carriage of the Apolipoprotein E-ε4 allele did not influence the degree of coronary stenosis. Group differences in heart weight remained significant after adjustment for age, gender, body mass index and apolipoprotein E genotype while differences in coronary artery stenosis were significantly associated with body mass index alone.

**Conclusions:**

The results are in agreement with an emerging understanding that, while midlife risk factors for ATH increase the risk for the later development of AD, once dementia begins, both risk factors and manifest disease diminish, possibly due to progressive weight loss with increasing dementia as well as disease involvement of the brain's vasomotor centers.

## Background

Alzheimer's disease (AD) is the most common neurodegenerative brain disease and the most common cause of dementia in the elderly. The disease has a progressive course over several years that often leaves the victim uncommunicative and bedridden. Five million Americans have AD and the cost of the disease to the US is estimated at $100 billion annually. Finding a cure or prevention for AD is an important national goal. Over the last decade considerable evidence has accumulated supporting a statistical linkage between atherosclerotic vascular disease (ATH) and AD. Several established risk factors for atherosclerosis, including hypertension, hypercholesterolemia, diabetes, cigarette smoking and the apolipoprotein E- ε4 allele have also been found to be risk factors for the development of AD [1-3]. This has important implications since major complications of ATH such as myocardial infarction and stroke are to a significant degree preventable through lifestyle modification and medications. If ATH and AD share aspects of their pathogenesis, AD may also be to some extent preventable through similar measures.

The relationship between AD and ATH is complex, however, and not completely understood. While elevated midlife ATH risk factors appear to increase the risk for developing AD in late life [4-6], once dementia begins, these same risk factors may diminish, perhaps due to molecular changes associated with AD, to decreased caloric intake and/or increased caloric expenditure or to reduced survival of those with high ATH risk factors [7-9].

The majority of the evidence linking AD and ATH has come from epidemiological and clinical studies. These are compromised by the inaccuracy of the clinical diagnosis of AD [10] and by the mostly indirect determinations of the presence and extent of ATH. Autopsy studies are a powerful method for investigating this issue as they offer a high diagnostic accuracy for AD and direct and accurate measures of ATH. To date, autopsy studies have reported a strong relationship between intracranial ATH and AD [11-13] while the correlation with coronary artery disease (CAD) has been less clear. Some groups have found a strong association [14-16] while others have failed to find an association [17,18]. A common limitation of these studies has been that the assessment of the degree of CAD has been solely through gross semi-quantitative estimates of the extent of stenosis, done routinely at autopsy and not specifically intended for research use. In this report, coronary artery stenosis in 57 deceased human subjects with and without a neuropathological diagnosis of AD was quantitatively assessed at multiple points along the coronary arterial system using computer-assisted image analysis. These measures, as well as measures of myocardial infarction volume, heart weight, ventricular wall thickness, valve circumferences and valve calcifications, were compared in AD and non-demented age-similar control subjects. Additionally, the prevalence of clinically-reported cardiovascular conditions and the apolipoprotein E (apoE) genotype were compared in the two groups.

## Methods

The study took place as part of the Banner Sun Health Research Institute Brain and Body Donation Program, which is a longitudinal, clinicopathologic study of normal aging and neurodegenerative disease in volunteers derived primarily from the surrounding retirement communities of Sun City, Sun City West, and Sun City Grand, Arizona [19]. The Program is approved by the Institutional Review Board of Banner Health, a regional, not-for-profit health care provider. Written informed consent was obtained from all subjects or their legal representatives.

Standardized clinical assessments were performed on most subjects every one to two years and at a minimum included a medical and family history questionnaire, height and weight measurements, the Folstein Mini-Mental State Examination (MMSE), one or more functional cognitive assessments and a neuropsychological test battery consisting of specific elements relating to attention, memory, language, visuospatial function and frontal/executive function. Dementia was diagnosed according to DSM-IV criteria, requiring an impairment of memory with one other abnormality of cognition, functional disability, and preservation of consciousness.

The neuropathological diagnosis of AD was made according to standardized criteria (National Institute on Aging/Reagan Institute "intermediate" or "high" probability") [20]. Inclusion criteria specified subjects whose autopsy had included the heart and had either a neuropathological diagnosis of AD or who had been clinically non-demented. Exclusion criteria specified subjects with a cause of dementia other than AD, including vascular dementia. Global cerebral senile plaque and neurofibrillary tangle load scores were obtained by summation of separate semi-quantitative density estimates of none, sparse, moderate or frequent (converted to 0-3 for statistical purposes) using standardized published templates [21]. Regions scored included cortical gray matter from frontal, parietal, temporal, hippocampal and entorhinal regions.

The epicardial coronary arteries were dissected out and then both arteries and hearts were fixed in formalin at 4 degrees C for 10 days followed by storage in phosphate-buffered saline with 0.1% sodium azide. The right coronary artery, marginal branch, right posterior descending, left coronary artery, circumflex artery and left anterior descending artery were cut every 5 mm into cross-sections that were examined using a Leica S8APO dissecting microscope. Digital photographs of the cross-sections were taken and measurements of the arteries' external and luminal areas were calculated using a desktop minicomputer with dedicated software (ImagePro Express, v.4.0; Media Cybernetics, Silver Spring, MD). An index of stenosis was calculated for each cross-section by subtracting the intraluminal area from the total area, dividing the difference by the total area and multiplying the quotient by 100. Hearts with surgical coronary artery bypasses (4 control subjects and 3 AD subjects) were excluded from the analysis.

Other heart measurements included heart weight, left and right ventricular wall thickness, valvular circumferences, presence or absence of valvular calcification and presence and volume of myocardial infarctions (MI). To assess the last-named, the left and right ventricles were sliced axially at 1 cm intervals and the number and volume of grossly-visible myocardial scars determined.

The Body Mass Index (BMI) was calculated for each subject from the last recorded height and weight measurements prior to death. ApoE genotyping was done with postmortem brain tissue using a standard technique [22].

Medical records from the subjects' private physicians were reviewed to record the number of cardiovascular risk factors and disease conditions that had been clinically diagnosed. These were grouped into the following categories: hypertension, hyperlipidemia/dyslipidemia, diabetes mellitus (type II), CAD, myocardial infarction, carotid artery disease, congestive heart failure, stroke/transient ischemic attack (TIA), valvular disease, peripheral arterial disease and arrhythmia. The timespan covered by the medical records available for review was also recorded.

Medication history is obtained from subjects at the time of their scheduled clinical research assessments. The analysis included all cardiovascular-related medications, grouped by medication class, that had been taken on the final clinical assessment. The medication classes were designated as anti-angina, anti-arrhythmia, anti-platelet, anti-diabetes, anti-hypertensive and lipid-lowering agents.

Statistical tests included unpaired, two-tailed t-tests to compare group means for individual measures, chi-square tests and Fisher's Exact tests for proportional differences, and logistic regression analysis to assess the independent effects on cardiovascular pathology measures of diagnosis, age, gender, BMI and apoE genotype. The significance level for all tests was set at 0.05.

## Results

General characteristics of the two groups are presented in Table 1. The mean ages did not differ (83.7 for the AD group and 84.4 for the control group). As expected, the mean MMSE score was significantly lower in the AD group. The mean BMI was also significantly lower in the AD group (23.2 vs 26.2 for controls). The intervals between the final MMSE and BMI determinations and death were not significantly different between the two groups. Measures of both senile plaques and neurofibrillary tangles were significantly greater in the AD group; the AD group had significantly lower mean brain weight and a significantly higher proportion of subjects carrying the apoE-ε4 allele.

**Table 1:** General characteristics of the study subjects.

Characteristic	AD N = 35	C N = 22
Age (years)	83.7 (6.6)	84.4 (6.0)
Gender (M:F)	16:19	15:7
BMI	23.2 (4.0)^2^	26.2 (4.1)
MMSE^1^	10.40 (7.93)^2^	28.53 (1.61)
Senile plaque density score	2.8 (0.38)^2^	1.4 (1.3)
Neurofibrillary tangle density score	4.6 (1.2)^2^	2.8 (0.91)
Brain weight (g)	1093 (132)^3^	1209 (101)
Number and percentage with the ApoE ε4 allele	15 (42.8%)^3^	4 (18.2%)

1. MMSE not available for 5 AD and 2 control subjects.

2. Significantly different from control (p < 0.01).

3. Significantly different from control (p < 0.05).

Means and standard deviations are shown. AD = Alzheimer's disease; C = non-demented elderly controls; BMI = body mass index; MMSE = mini mental state examination; ApoE = Apolipoprotein E.

The prevalence of clinically documented cardiovascular risk factors and disease conditions in the diagnostic groups is shown in Table 2. The mean number of months covered by the available private medical records did not significantly differ between groups. Subjects with AD had significantly fewer total recorded conditions than control subjects (2.91 vs 4.18; p < 0.01) and had lower prevalences of each of the condition categories recorded except for stroke/transient ischemic attack for which there was a higher prevalence in the AD subjects (45.7% vs 27.3%; p = 0.13). Of individual condition categories, only the prevalence of congestive heart failure was significantly different between the diagnostic groups (2.9% vs 36.4% for AD and controls, respectively; p < 0.01). There was a trend for peripheral vascular disease to be less common in AD (8.6% vs 27.3%; p = 0.07). For every medication class except anti-platelet agents, a smaller proportion of AD subjects were taking the agents (Table 3). The proportional differences were significant for anti-hypertensive agents (25.9% of AD subjects vs 71.4% for control subjects).

**Table 2:** Clinically documented cardiovascular risk factors and disease conditions amongst study subjects.

Condition	AD	Control
Hypertension	22/35 (62.8%)	18/22 (81.8%)
Hyperlipidemia/Dyslipidemia	14/35 (40%)	12/22 (54.5%)
Diabetes mellitus	7/35 (20%)	7/22 (31.8%)
Coronary Artery Disease	15/35 (42.9%)	11/22 (50%)
Myocardial infarction	5/35 (14.3%)	5/22 (22.7%)
Arrhythmia	13/35 (37.1%)	13/22 (59.1%)
Valvular disorder	2/35 (5.7%)	3/22 (13.6%)
Congestive heart failure	1/35 (2.9%)^1^	8/22 (36.4%)
Carotid artery disease	2/35 (5.7%)	3/22 (13.6%)
Stroke/Transient ischemic attack	16/35 (45.7%)	6/22 (27.3%)
Peripheral vascular disease	3/35 (8.6%)	6/22 (27.3%)
Total Number of Conditions	2.91 (1.64)^1^	4.18 (1.84)
Timespan covered by records (months)	73.5 (50.8)	82.1 (73.4)

1. Significantly different from control (p < 0.01).

Values shown are proportions and percentages affected within each diagnostic group, except for the total number of conditions and the timespan covered by the available medical records, where the means and standard deviations are shown.

**Table 3:** Proportion and percentage of subjects taking cardiovascular-related medication types at the last clinical assessment prior to death.

Medication Class	Alzheimer's	Control
Anti-angina	0 (0%)	1 (4.8%)
Anti-arrhythmia	2 (7.4%)	2 (9.5%)
Anti-platelet	13 (48.1%)	7 (33.3%)
Anti-diabetic	2 (7.4%)	5 (23.8%)
Anti-hypertensive	7 (25.9%)*	15 (71.4%)
Lipid-lowering	5 (18.5%)	6 (28.6%)

* p < 0.05, Fisher's exact test

There were no significant differences between groups except for the proportion taking anti-hypertensive agents. Data was available for 27 AD subjects and 21 control subjects.

**Figure 1:**
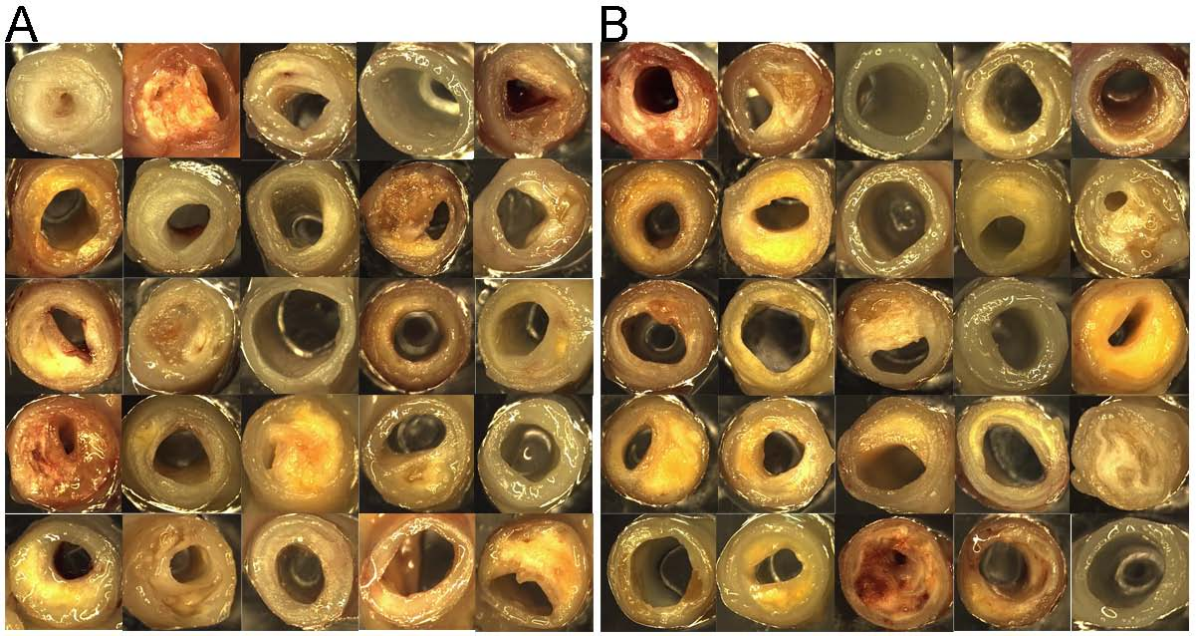
**Representative digital images of coronary artery cross-sections, depicting the range of atherosclerotic stenosis amongst study subjects**. Arteries from control subjects are shown in (A) while arteries from AD subjects are shown in (B).

**Figure 2:**
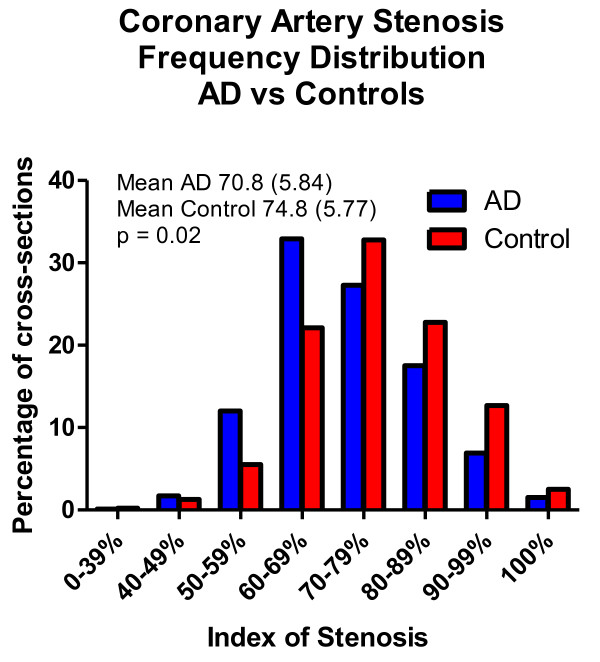
**Class frequency histogram comparing the index of stenosis in coronary artery disease cross-sections from AD (blue) and control (red) groups**. The means and standard deviations for all cross-sections from AD and control groups are also shown; the difference was significant (p = 0.02).

Figure 1 depicts gross photographs of representative coronary artery cross-sections, displaying the range of atherosclerotic stenosis observed. Differences between the two groups were not qualitatively apparent. As the index of stenosis within the diagnostic groups was not significantly different between different coronary artery branches, between males and females or on the basis of carriage of the apoE-ε4 allele, the data from all coronary branches and all subjects was combined for further comparison of the diagnostic groups. Figure 2 graphically compares the mean index of stenosis in coronary arteries from AD and control subjects. The graph is a class-frequency histogram, with the height of the columns being proportional to the percentage of cross-sections with a particular index of stenosis range. The entire histogram is shifted to the right for control subjects, indicating a higher proportion of cross-sections with a higher index of stenosis. The means for all cross-sections are also shown for the two groups; these differed significantly, with a greater mean index of stenosis in the control group (74.8 vs 70.8; p = 0.02).

A greater proportion of control subjects had myocardial infarctions (50% vs 28.6%) but this did not meet the significance level (p = 0.10). The mean MI volume was higher in control subjects than in AD subjects (3.56 vs 1.65 cm^3^) but the again the difference was not significant.

Other heart measurements including heart weight, left and right ventricle mean thickness, and valve circumferences are presented in Table 4, grouped by diagnosis and gender. For both males and females, the control group had greater mean heart weights as well as greater mean tricuspid and mitral valve circumferences. The difference in heart weight was particularly striking, with mean heart weights from male and female control subjects being 87 and 93 grams heavier than those from male and female AD hearts, respectively. The difference between female control and AD heart weights was significant (p < 0.05) while the difference between male heart weights was close to the significance level (p = 0.08). Mitral and tricuspid valve circumferences were significantly greater in female control subjects as compared to female AD subjects (p < 0.01). Ventricular thickness did not differ significantly between the groups but there was a trend for thicker left and right ventricular walls in the control group for both genders. Body mass index was higher in the control group for both genders (Table 4) and the difference in means reached the significance level for females (p < 0.01) but not for males (p = 0.09).

**Table 4:** Comparison of heart weight, ventricular thickness, and valve circumferences, with respect to diagnosis and gender.

Category	AD Male	Control Male	AD Female	Control Female
Heart Weight (g)	402 (131)	489 (136)	319 (99)^1^	412 (69)
Mean LV greatest thickness (cm)	1.4 (0.19)	1.51 (0.27)	1.4 (0.21)	1.43 (0.39)
Mean RV greatest thickness (cm)	0.46 (0.05)	0.48 (0.10)	0.40 (0.08)	0.47 (0.13)
BMI	23.9 (4.00)	26.5 (3.81)	22.6 (3.94)^1^	28.4 (4.67)
Tricuspid valve circumference (cm)	11.21 (1.70)	11.49 (1.59)	9.9 (1.04)^2^	11.5 (1.32)
Pulmonary valve circumference (cm)	9.15 (0.90)	8.23 (0.96)	7.45 (0.90)	7.81 (0.98)
Mitral valve circumference (cm)	10.49 (1.83)	10.89 (0.94)	8.95 (0.87)^2^	10.26 (1.44)
Aortic valve circumference (cm)	9.86 (1.11)	8.93 (1.17)	8.15 (0.86)	7.5 (0.50)

1. Significantly different from female control subjects (p < 0.05).

2. Significantly different from female control subjects (p < 0.01).

Means and standard deviations are shown. LV = left ventricle; RV = right ventricle; BMI = body mass index

Tricuspid valve calcification was uncommon and there were no subjects with pulmonary valve calcification. Mitral and aortic valve calcification was present in 50% to 68% of subjects. The proportional differences between groups did not differ significantly for any of the valves.

Logistic regression analysis was performed to further investigate the two major findings, i.e. decreased heart weight and decreased coronary artery stenosis in AD subjects. Assignment of greater pathology status was based on measurements above the median value for all subjects. The analysis was primarily for the effect of diagnostic status as AD or control and was adjusted for age, gender, apoE genotype and BMI. Diagnostic status had a significant effect (p < 0.05, Wald p-value) on heart weight. None of the other variables had a significant association with heart weight but BMI was close to the significance level (p = 0.067, Wald p-value) and thus may have been significant with a larger subject number. Controls were 4.47 times more likely (OR = 4.47) to have increased heart weights. For the analysis of coronary artery stenosis, diagnostic status had no significant effect while BMI was the sole significant variable (p < 0.05, Wald p value). Each point increase in BMI was associated with a 1.24% increase in coronary artery stenosis (OR = 1.24).

## Discussion

The "vascular hypothesis" of AD is as old as the disease itself, as many early investigators assumed that AD was caused by cerebral atherosclerosis. Others, including Alzheimer himself, believed that the two processes were unrelated. The relationship between AD and cerebrovascular disease was eventually revised to the point of complete separation due to a series of postmortem investigations during the 1950's and 1960's [23].

The discovery in 1993 of the association of the apoE-ε4 allele with AD [24] revived interest in the vascular hypothesis as the ε4 allele was already known to be associated with coronary atherosclerosis. Subsequent epidemiologic and clinical investigations have established that multiple additional ATH risk factors are also risk factors for AD [1-3]. There have been relatively few confirmatory autopsy studies but the published reports to date indicate a strong relationship between intracranial ATH and AD [11-13,25] while the correlation with CAD has been less clear [14-18]. The present study has utilized precise coronary artery stenosis measurements and neuropathologically examined subjects in an attempt to provide more clarity on this subject. Additionally, measures of general heart pathology have been compared.

The results show a clear tendency for better cardiovascular health in the AD group. Of ten clinically documented cardiovascular risk factors or disease conditions, nine occurred more frequently in the control group. Only stroke/TIA was more frequent in the AD subjects. In terms of gross anatomy, the control group had greater coronary artery stenosis, a greater proportion with myocardial infarcts, larger myocardial infarcts, greater heart weights, thicker ventricular walls and greater triscuspid and mitral valve circumferences. For every medication class except anti-platelet agents, a smaller proportion of AD subjects were taking the agents, making it unlikely that these findings were due to increased anti-cardiovascular disease treatment in AD subjects.

Previous autopsy studies of the heart in AD are relatively scarce but are mostly in agreement with the present study. Heart weights, in particular, have been reported to be lower in AD in two previous studies [15,26]. One previous study has reported less coronary artery stenosis in subjects with AD [17]. Two studies by another group [18,27] did not report directly on coronary artery atherosclerosis but instead used a cardiovascular pathology index (CVI) composed of several gross anatomical measures including heart weight, coronary artery atherosclerosis, myocardial infarctions, peripheral artery atherosclerosis and intracranial atherosclerosis. Their results showed less cardiovascular pathology in AD and an inverse correlation with the densities of the histopathologic lesions of AD. One group reported mildly increased CAD in AD subjects [15]. Possible reasons for the discrepancy between this last study and the present report are the smaller size and lower BMI of the control group in the previous study (N = 12; BMI = 23.0) as compared to the present study (N = 22; BMI = 26.2). It is possible that the method of assessing CAD differed between the two studies but as this was not described in the previous study no conclusions can be made regarding this issue. To our knowledge, the present study is the first to use precise, computer-assisted image analysis to determine the degree of coronary artery stenosis in AD and control subjects.

Although the apoE-ε4 allele has been reported to be associated with clinical CAD, the present findings are in agreement with studies finding no association. In terms of the interaction of the ε4 allele with both coronary atherosclerosis and AD, one study has reported that the ε4 allele correlated with increased coronary atherosclerosis within AD subjects [26] but did not compare coronary atherosclerosis in AD subjects with that in control subjects. Another study reported that the degree of coronary atherosclerosis correlated with the density of AD-related histopathologic lesions in subjects carrying the ε4 allele [15].

The results of this study support an emerging body of data consistent with decreased cardiovascular risk factors and disease conditions in elderly subjects with AD [27-29]. This has been somewhat surprising as authoritative reports from groups conducting population-based longitudinal studies have indicated that ATH risk factors including hypertension and hypercholesterolemia, when present in midlife, are strongly predictive for the development of AD in late life [4-6]. It has been postulated that the reversal of cardiovascular pathology in elderly AD subjects may arise due to molecular or neuropathological factors [7-9] associated with AD. For example, brainstem neurons that contribute to vasomotor regulation are lost in AD and this could result in decreased blood pressure and heart weight. Although we did not have longitudinal data confirming progressive weight loss over time in our AD subjects, this has been documented in several longitudinal studies [30-33] and therefore it seems likely that this plays a major role as well.

## Conclusions

The present study has found consistently decreased measures of both clinical and gross anatomic indices of cardiovascular disease, including coronary artery stenosis and cardiac hypertrophy, in elderly AD subjects as compared with age-similar non-demented subjects. The reasons for this are uncertain but may be due to progressive weight loss in AD subjects as well as to disease-specific effects on brain vasomotor regulatory neurons.

## Competing interests

The authors declare that they have no competing interests.

## Authors' contributions

All authors have read and approved the final manuscript. TGB and AER contributed to conception and design as well as to analysis and interpretation of data and were involved in drafting the manuscript and revising it for important intellectual content. CLM, RGB, SS, IDD, LIS, MNS, DGW and LL all contributed to acquisition and/or analysis of data.

## Pre-publication history

The pre-publication history for this paper can be accessed here:

http://www.biomedcentral.com/1471-2318/11/3/prepub
